# Personal protective equipment in the COVID-19 pandemic and the use of cooling-wear as alleviator of thermal stress: A pilot study in plastic surgery staff members

**DOI:** 10.1007/s00508-020-01775-x

**Published:** 2020-12-10

**Authors:** Hanna Luze, Sebastian P. Nischwitz, Petra Kotzbeck, Julia Fink, Judith C. J. Holzer, Daniel Popp, Lars-Peter Kamolz

**Affiliations:** 1 JOANNEUM RESEARCH Forschungsgesellschaft mbH, COREMED—Cooperative Centre for Regenerative Medicine, Neue Stiftingtalstraße 2, 8010 Graz, Austria; 2 Department of Surgery, Division of Plastic, Aesthetic and Reconstructive Surgery, Medical University of Graz, Graz, Austria

**Keywords:** Thermal stress, Cooling wear, COVID-19, PPE, Concentration

## Abstract

**Background:**

High temperatures at workplaces lead to health-related risks and premature exhaustion. The coronavirus disease 2019 (COVID-19) pandemic requires many health professionals to perform under unfavorable conditions. Personal protective equipment (PPE) causes thermal stress and negatively affects performance.

**Patients, materials and methods:**

This pilot project investigated the effects of PPE and additional cooling wear on physiological parameters and concentration of six healthy staff members of the Plastic Surgery Department of the Medical University of Graz, Austria during simulated patient care. In this study two 1‑hour cycles with patient care-related tasks with PPE and PPE + cooling-wear, respectively, were conducted. A third cycle with scrubs exclusively served as baseline/negative control. The assessment occurred immediately pre-cycles and post-cycles.

**Results:**

Pre-cycle assessments showed no significant differences between the cycles. After PPE cycle, increased physical stress levels and decrements in concentration capacity were observed. Physiological parameters were significantly less affected in the cooling cycle, while concentration capacity slightly increased.

**Conclusion:**

COVID-19 PPE causes considerable thermal stress, ultimately affecting human performance. As opportunity to withstand thermal stress, and improve patients’ and professionals’ safety, cooling-wear can be considered relevant. Medical personnel performing in exceptional situations may particularly benefit from further development and investigation of cooling strategies.

## Introduction

Several professions require the performance in high-temperature scenarios leading to physical stress, ultimately affecting human performance [1]. For instance, medical personnel in the care of patients suffering from potentially dangerous and infectious diseases need to use personal protective equipment (PPE) causing considerable discomfort [2]. The novel severe acute respiratory syndrome-coronavirus‑2 (SARS-CoV-2) and the resulting coronavirus disease 2019 (COVID-19) pandemic have become a serious challenge regarding spread, treatment, and prevention [3]. Involved caregivers need to work with impermeable PPE, consisting of FFP3 face masks, protective goggles, gloves and suits, to avoid contagion while ensuring proper medical care [4, 5]. Effective shielding is not only necessary for healthcare workers and patients, to maintain adequate healthcare but also the whole population by reducing further spreading of pathogens [6]. Besides the morbidity among patients [7], SARS-CoV‑2 also mediates higher morbidity among medical staff [5]. Not only psychological stress and anxiety [5] but also high temperatures resulting in thermal stress caused by PPE should be considered a major risk factor.

While physiological responses of the human organism to high temperatures have been well established, their impact on cognition and well-being is less clear [9]; yet, even relatively mild thermal stress may affect human performance [1, 8]. Considerable discomfort and medical negligence can be effects thereof [10], hence negatively influencing a patient’s care.

Only few studies have evaluated different PPEs or ways of donning and doffing [2]. A significant heat strain has been shown with selectively permeable membranes [11]. Considering that fluid-resistant and impermeable suits may protect better against contamination than aprons [2], people caring for COVID-19 patients suffer from the additional burden of significant thermal stress when using effective protection.

The use of cooling wear is one novel strategy using the principle of evaporation to reduce thermal stress in situations exposing professionals to high temperatures [12]. While experiences with cooling wear in the medical field showed positive results in previous studies [13], the benefit of use in combination with PPE still needs to be determined. The primary aim of this project was to investigate the impact of PPE used in the treatment of COVID-19 patients on the human organism as well as the effect of additional cooling wear as a suitable coping strategy.

## Methods

The study design and protocol were approved by the ethical review committee of the Medical University of Graz (EK: 1318/2019). Written informed consent was obtained from study participants prior to the study start.

### Participants

In this study four female (age range: 27–40 years) and two male (range: 30–48 years) healthy, non-pregnant staff members of the Plastic Surgery Division of the University Hospital Graz, Austria, were enrolled.

### Cooling wear

The tested cooling wear QRCOOL was provided by QRSKIN GmbH (Würzburg, Germany) [14].

### Experimental design

The study was conducted under constant regular room temperature (22 °C ± 0.5 °C) at the University Hospital Graz. The study consisted of three cycles that were performed within 2 days:

1. Cycle one (base cycle) served as a baseline. Subjects’ garment consisted of regular scrubs exclusively to determine effects of the tasks to perform.
2. Cycle two (PPE cycle) served to explore effects of PPE. Subjects’ garment consisted of scrubs plus PPE as used in the care of COVID-19 patients (suits: Tychem 2000 C Cat. III model CHA5, DuPont de Nemours, Wilmington, DE, USA, 180 μm thickness and model FH-190, Beijing Winsunny Harmony Science & Technology Co., Ltd., Beijing, China, 240 µm thickness).
3. Cycle three (cooling cycle) served to explore effects of PPE and additional cooling-wear. Subjects’ garment consisted of a cooling shirt/vest, which was worn on bare skin, plus scrubs and PPE.

Subjects were instructed about the process, completed a standardized d2‑R attention test [15], underwent thermal imaging of chest and upper back and had blood pressure (BP), heart rate (HR) and body temperature (BT) measured before each cycle. All assessments were performed with the garment of each respective cycle. Afterwards, subjects performed standardized tasks for 1h. A total of six tasks representing abilities needed in COVID-19 patient care, targeting mental capacity (e.g. logic-based puzzles), manual dexterity (e.g. surgical sewing) and strength (e.g. static strain) lasting 9 min each had to be completed by each subject. A rotation time of 1 min was determined between tasks. Fig. 6 in the Appendix depicts the schematic course of the activity cycles. After each cycle, subjects underwent the same assessment procedures again before having an adequate break. Subjects then proceeded to the second cycle undergoing the same procedure with scrubs and PPE. To rule out an effect of fatigue or familiarization, the third cycle (cooling cycle) was performed on the next day with the otherwise same set-up.

### Measurements

#### Thermal imaging

Thermal imaging was conducted with FLIR One® Pro for iOS (FLIR Systems, Wilsonville, OR, USA). Imaging material was processed via FLIR One® App [16]. Within a region of interest (ROI, the human body), the average temperature was calculated.

#### Body temperature

The BT was measured with a contactless infrared forehead thermometer (Hetaida HTD8812, Shenzhen Hetaida Technology Co., Ltd., China).

#### Blood pressure and heart rate measurement

Systolic and diastolic BP, and HR were measured with a noninvasive, fully automatic BP monitor (boso-medicus uno, BOSCH + SOHN GmbH u. Co. KG, Jungingen, Germany).

#### Standardized concentration and attention test

Concentration and attention were measured with the d2‑R test of attention, which is a reliable measure of attention based on normative samples [15]. The total number of errors (TNE) and the concentration performance indicator (CPI) were calculated.

### Statistical analysis

Data were analysed with GraphPad Prism software (version 8.0; GraphPad Software, Inc., San Diego, CA, USA). Due to the small sample size, testing for normality was omitted. Since the conditions for the parametric t‑test could not be guaranteed, group differences were assessed using the non-parametric Wilcoxon signed-rank test. Results are expressed as median (mdn) and interquartile range (Q1 and Q3). Mann-Whitney U‑test was used to determine significant differences between the two types of protective clothing. Results in means and standard deviations are summarized in Table 1 in the Appendix. All statistical tests were two-tailed with the alpha level set at *p* = 0.05.

## Results

**Fig. 1:**
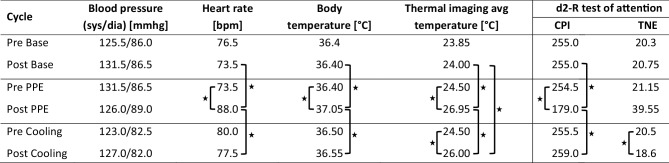
Measurements. Descriptive overview of BP, HR, body temperature, average thermal imaging temperature, concentration performance indicator (CPI) and total number of errors (TNE). The mdn values before and after the activity are shown. *An asterisk* indicates statistical significance. *avg* average, *bpm* beats per minute, *CPI* concentration performance indicator, *dia* diastolic, *sys* systolic, *TNE* total number of errors

A descriptive overview of all physiological parameters is provided in Fig. 1.

### Body temperature

**Fig. 2:**
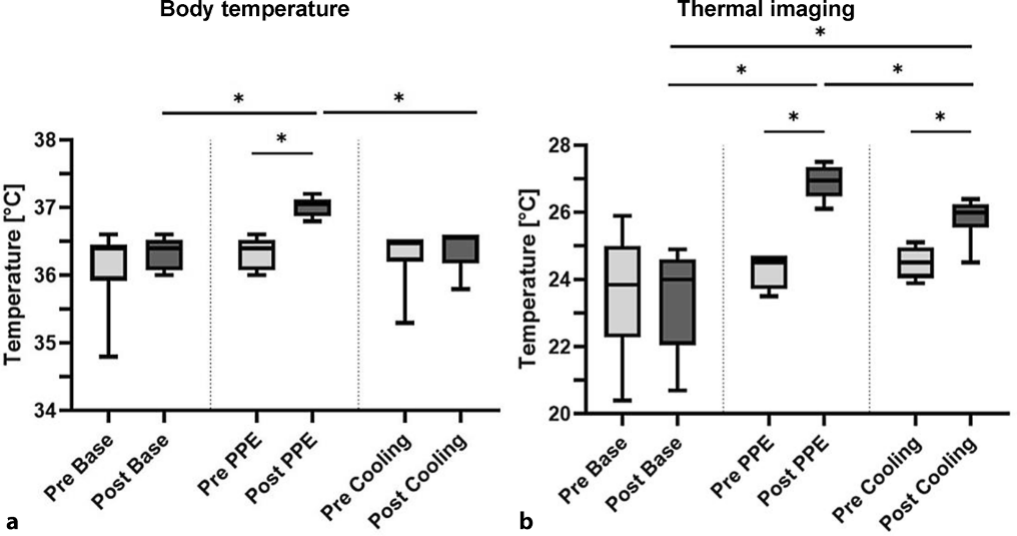
Results of the body temperature (BT) measurements and thermal imaging. **a** Body temperature. **b** Thermal imaging. BT measurements and thermal imaging were performed before and after the activity period. In contrast to pre-cycle levels, a significant increase of BT (*p* = 0.031) and surface temperature (*p* = 0.031) was shown post-PPE cycle. Boxplots represent median, quartiles and min–max values. An *asterisk* indicates statistical significance

The BT of each subject was measured pre-cycle and post-cycle. Fig. 2 shows boxplots of the BT results.

#### Base cycle

Median BT before the base cycle was 36.40 °C (Q1: 35.93 °C; Q3: 36.45 °C). After completion it was 36.40 °C (Q1: 36.08 °C; Q3: 36.53 °C). No significant difference was observed (*p* = 0.875).

#### PPE cycle

Median BT before the PPE cycle was 36.40 °C (Q1: 36.08 °C; Q3: 36.53 °C). It rose to 37.05 °C after the cycle. The difference before and after the cycle was significant (*p* = 0.031).

#### Cooling cycle

Median BT before the cooling cycle was 36.50 °C (Q1: 36.20 °C; Q3: 36.50 °C). After the cycle, it rose to 36.55 °C (Q1: 36.18 °C; Q3: 36.60 °C). The difference before and after the cycle was not significant (*p* = 0.500).

#### Base vs. PPE cycle

The pre-cycle assessment showed no significant difference in median BTs (*p* = 0.875). The difference after the two cycles was significant (*p* = 0.031).

#### Base vs. cooling cycle

The pre-cycle assessment showed no significant difference in median BTs (*p* = 0.156). The difference after these two cycles was not significant (*p* = 0.875).

#### PPE vs. cooling cycle

The pre-cycle assessment showed no significant difference in median BTs (*p* > 0.999). The difference after these two cycles was significant (*p* = 0.031).

#### Suit comparison

Two different protective suits were analyzed regarding their heat production potential. No significant difference could be shown in BT between the suits neither within nor between PPE (*p* = 0.500) and cooling cycle (*p* = 0.100).

### Thermal imaging

**Fig. 3:**
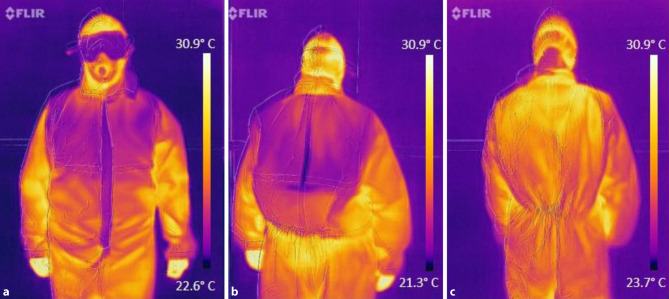
Thermal imaging via FLIR One® Pro. **a** Front image of a study participant with cooling wear and PPE after the cooling cycle. **b** Back image of a study participant with cooling wear and PPE after the cooling cycle. **c** Back image of a study participant with PPE after the PPE cycle (no cooling wear). Images (**a**) and (**b**) show a distinct cooling effect after the activity cycle in the region of the cooling wear

Surface temperature (ST) of each subject was measured via FLIR One® Pro pre-cycle and post-cycle. Fig. 2 shows boxplots of the thermal imaging results. Cooling effects can be seen on chest and upper back after cooling cycle (see Fig. 3).

#### Base cycle

Median ST before base cycle was 23.85 °C (Q1: 22.28 °C; Q3: 25.00 °C). Afterwards, it was 24.00 °C (Q1: 22.05 °C; Q3: 24.60 °C). No significant difference was observed (*p* = 0.656).

#### PPE cycle

Median ST before PPE cycle was 24.50 °C (Q1: 23.73 °C; Q3: 24.70 °C). Afterwards, it rose to 26.95 °C (Q1: 26.48 °C; Q3: 27.35 °C). The difference was significant (*p* = 0.031).

#### Cooling cycle

Median ST before cooling cycle was 24.50 °C (Q1: 24.05 °C; Q3: 24.95 °C). Afterwards, it rose to 26.00 °C (Q1: 25.55 °C; Q3: 26.25 °C), indicating a significant difference (*p* = 0.031).

#### Base vs. PPE cycle

Pre-cycle assessment showed no significant difference in median ST (*p* = 0.688). The difference thereafter was significant (*p* = 0.031).

#### Base vs. cooling cycle

Pre-cycle assessment showed no significant difference in median ST (*p* = 0.219). The difference after these cycles was significant (*p* = 0.031).

#### PPE vs. cooling cycle

The pre-cycle assessment showed no significant difference in median ST (*p* = 0.188). The difference after these cycles was significant (*p* = 0.031).

### Heart rate

**Fig. 4:**
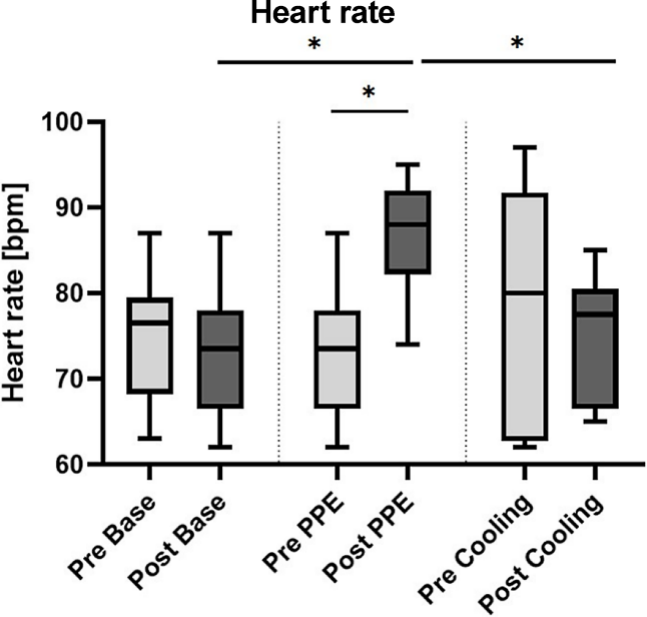
Results of the heart rate (HR) measurements. HR of each subject was measured before and after the testing period. A significant increment of the HR was found in the PPE cycle compared to pre-cycle levels. HR after the cooling cycle was significantly lower than after the PPE cycle (*p* = 0.031). Boxplots represent median, quartiles and min-max values. An* asterisk* indicates statistical significance

The HR of each subject was measured pre-cycle and post-cycle. Fig. 4 shows boxplots of the heart rate results.

#### Base cycle

Median heart rate before the base cycle was 76.5 bpm (Q1: 68.3 bpm; Q3: 79.5 bpm) and 73.5 bpm (Q1: 66.5 bpm; Q3: 78.0 bpm) afterwards, yielding no significant difference (*p* = 0.063).

#### PPE cycle

Median heart rate before the PPE cycle was 73.5 bpm (Q1: 66.5 bpm; Q3: 78.0 bpm), afterwards it rose to 88.0 bpm (Q1: 82.3 bpm; Q3: 92.0 bpm), yielding a significant difference (*p* = 0.031).

#### Cooling cycle

Median heart rate before the cooling cycle was 80.0 bpm (Q1: 62.8 bpm; Q3: 91.8 bpm), afterwards it decreased to 77.5 bpm (Q1: 66.5 bpm; Q3: 80.5 bpm), yielding no significant difference (*p* = 0.406).

#### Base vs. PPE cycle

The pre-cycle assessment showed no significant difference in median HR (*p* = 0.063). The difference after the cycles was significant (*p* = 0.031).

#### Base vs. cooling cycle

The pre-cycle assessment showed no significant difference in median HR (*p* = 0.500). The difference after the cycles was not significant (*p* = 0.406).

#### PPE vs. cooling cycle

The pre-cycle assessment showed no significant difference in median HR (*p*-value = 0.313). The difference after the cycles was significant (*p* = 0.031).

### Blood pressure

Systolic and diastolic BP were measured pre-cycle and post-cycle. No significant differences either before and after or between the cycles were shown.

### Concentration test

**Fig. 5:**
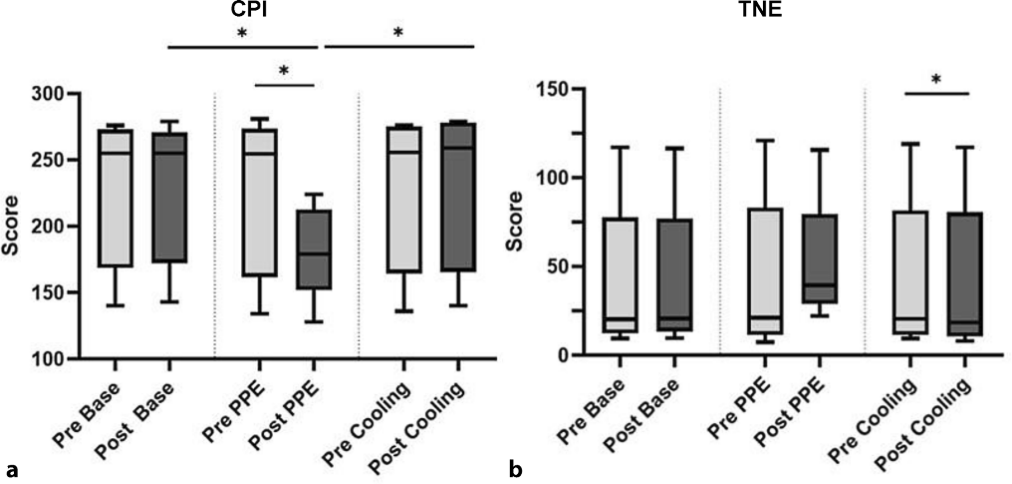
Concentration performance indicator and total numbers of errors. **a** CPI. Post-PPE cycle, CPI was significantly lower than post-base cycle and post-cooling cycle. **b** TNE. A significantly decreased TNE after the cooling cycle (*p* = 0.031) was shown. Boxplots represent median, quartiles and min–max values. An* asterisk *indicates statistical significance

Concentration performance indicator (CPI) and total number of errors (TNE) of each subject were determined pre-cycle and post-cycles. Test results are summarized in Fig. 5.

**Fig. 6:**
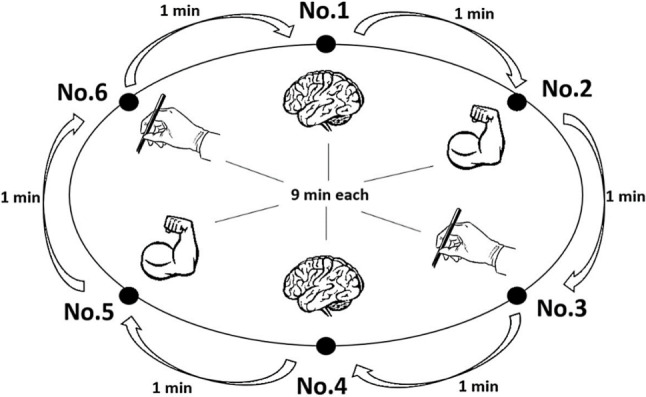
Schematic description of the study design. Six tasks targeting concentration (brain), manual dexterity (hand with surgical forceps) and strength (flexing arm) lasting 9 min each were completed. A 1-min rotation time was scheduled between the tasks

#### Base cycle

Median CPI and TNE before base cycle were 255.0 (Q1: 168.5; Q3: 273.0) and 20.3 (Q1: 12.4; Q3: 77.7), respectively. Afterwards, they were 255.0 (Q1: 172.3; Q3: 270.8) and 20.8 (Q1: 13.5; Q3: 77.2), respectively, indicating no significant difference (CPI: *p* = 0.594; TNE: *p* = 0.625).

#### PPE cycle

Median CPI and TNE before PPE cycle were 254.5 (Q1: 161.8; Q3: 273.5) and 21.2 (Q1: 11.5; Q3: 83.4), respectively. Afterwards, they were 179.0 (Q1: 152.0; Q3: 212.8) and 39.6 (Q1: 29.0; Q3: 79.7), respectively, indicating a significant difference for CPI (*p* = 0.031), but not TNE (*p* = 0.219).

#### Cooling cycle

Median CPI and TNE before cooling cycle were 255.5 (Q1: 164.5; Q3: 275.3) and 20.5 (Q1: 11.5; Q3: 81.7), respectively. Afterwards, they were 259.0 (Q1: 165.5; Q3: 278.3) and 18.6 (Q1: 10.7; Q3: 80.7), respectively, indicating no significant difference for CPI (*p* = 0.063), but for TNE (*p* = 0.031).

#### Base vs. PPE cycle

Pre-cycle assessments showed no significant differences between CPI (*p* = 0.344) or TNE (*p* = 0.563). After the cycles, CPI yielded a significant difference (*p* = 0.031), but not TNE (*p* = 0.063).

#### Base vs. cooling cycle

Pre-cycle assessments showed no significant differences for CPI (*p* = 0.813) or TNE (*p* = 0.563). Afterwards no significant differences were seen either (CPI: *p* = 0.750, TNE: *p* = 0.688).

#### PPE vs. cooling cycle

Pre-cycle assessments showed no significant difference between CPI (*p* = 0.656) or TNE (*p* = 0.594). Afterwards, the difference between CPI was significant (*p* = 0.031), but not between TNE (*p*-value = 0.156).

## Discussion

There is increasing concern about diverse stressors affecting physicians during various scenarios [17]. Thermal stress as caused by PPE is one well-documented factor decrementing vigilance and endurance [1, 10] and directly influencing human performance, whose flawless functioning is of utmost importance during exceptional situations such as the current COVID-19 pandemic. We could show that even relatively mild thermal stress negatively affects concentration, while increasing physiological stress levels. Parameters such as HR [17], BP or BT served as variables indicating stress levels, subjects were experiencing. Homogeneous values before and after base cycle proved that the performed tasks themselves do not cause an elevated physical stress level. After PPE cycle, significantly increased BT (*p* = 0.031) and HR (*p* = 0.031) were observed, suggesting considerable high physical (thermal) stress compared to pre-cycle levels.

Several studies investigated effects of heat on cognition [18, 19]. Our study could confirm the described correlation between increased temperatures and decrements in concentration and performance, since lower attention test results were produced after PPE cycle. Compared to the pre-cycle, CPI significantly decreased afterwards (*p* = 0.031). PPE may not only constitute an intense burden, but ultimately jeopardize adequate patient care due to its negative impact on the users’ performance. To ensure proper medical care of COVID-19 patients while providing highest possible comfort for medical personnel, achieving a balance between sufficient protection and reduced heat strain is of utmost importance. In order to achieve this goal, several aspects need to be considered. More breathable materials lead to higher user satisfaction as shown by Verbeek et al. [2]. Heat generation of less breathable materials could be a contributing factor for this, which is why two different suits differing in thickness were tested in our study. No significant difference regarding their heat production potential in the study period, neither after the PPE cycle (*p* = 0.500) nor after the cooling cycle (*p* = 0.100) was shown.

Cooling-wear may be an effective means to reduce thermal stress caused by PPE. Subjects wearing cooling-wear beneath scrubs and PPE were significantly less affected. This suggests the ability of cooling-wear to reduce the physiological stress level caused by increased temperatures. Physiological parameters after the cooling cycle almost reached pre-cycle levels. Not only BT (*p* = 0.031) but also ST (*p* = 0.031) was significantly lower after cooling compared to after PPE cycle. An increase of BT within cooling cycle was seen (24.50–26.00 °C); however, less pronounced than in PPE cycle (24.50–26.95 °C). Hereby, a counteracting function of cooling-wear against heat production below PPE is suggested. Even after 1h of activity, a visible cooling effect was revealed by thermal imaging (Fig. 3).

Furthermore, positive effects on concentration and attention were observed in subjects wearing cooling wear. CPI was significantly higher after cooling than after PPE cycle (*p* = 0.031), while a lower TNE, although not significant (*p* = 0.156), was observed. Ultimately, an improvement of cognitive performance in warm environments is supposedly possible with cooling-wear.

The small sample size may lead to not fully representative and transferable results; however, this novel invention might be able to bridge the gap between proper medical care without contagion and adequate comfort for medical personnel. Particularly, caregivers of COVID-19 patients may profit from further development and in-depth investigation of cooling strategies. Further studies are necessary to achieve the full potential of cooling-wear in reducing thermal stress.

### Limitations

This study has several limitations: We aimed to investigate the burden of PPE and the advantages of novel cooling strategies for use in the medical field. Due to the small sample size, results may not be representative and transferable. Further studies are necessary to reach the full potential of cooling-wear in reducing thermal stress. While the study set-up was designed to reduce the possible bias caused by fatigue and repetition, individual performance levels (cognitive and physical) are dependent on personal form of the day. This is for example reflected in higher pre-cycle HR, even though not statistically significant (pre-cooling: 80.0 bpm vs. pre-PPE: 73.5 bpm). Analysis of BP values revealed no significant differences. The values of each subject, however, varied strongly, especially post-PPE. This divergence indicates individual reactions of the circulatory system to thermal stress in each subject. Consequently, a bias by individual factors, such as daily activity or autonomic nerve tone, cannot be ruled out. Ultimately, there is a great demand for strategies to withstand thermal stress and reduce discomfort caused by PPE and to improve working conditions during these challenging times.

## Conclusion

Decrements in vigilance, performance and endurance are well-documented effects of thermal stress and have been confirmed in this study. Thermal stress due to PPE used in the management of COVID-19 patients leads to increased BT and HR. Simultaneously, the concentration capacity decreases and the error-proneness increases. These results highlight the burden of PPE, medical personnel have to bear during the current COVID-19 pandemic. As an opportunity to withstand thermal stress and therefore improve medical care, cooling strategies are useful in many aspects. Most physiological parameters measured after cooling cycle almost reached pre-cycle levels, indicating lower physiological stress levels. Due to the need of the balance between sufficient protection and reduced heat strain, cooling-wear serves as promising opportunity to counteract encumbering situations with heat exposure.

Based on the promising results obtained from this project, cooling strategies may be used as important tool in medical sectors prospectively. Further studies are necessary to achieve the full potential of this newly developed cooling-wear.

## Appendix

**Table 1:** Measurements. Descriptive overview of body temperature, average thermal imaging temperature, blood pressure, heart rate, concentration performance indicator and total numbers of errors

Cycle	Body temperature [°C]			Thermal Imaging avg. temperature [°C]			Blood pressure (sys/dia) [mm Hg]									Heart rate [bpm]			d2‑R test of attention					
																			CPI			TNE		
	Mean	SD	Median	Mean	SD	Median	Mean			SD			Median			Mean	SD	Median	Mean	SD	Median	Mean	SD	Median
Pre Base	36.15	0.67	36.40	23.60	1.88	23.85	126.50	/	86.00	7.87	/	8.64	125.50	/	84.83	75.00	8.03	76.50	229.30	55.67	255.00	40.88	42.42	20.30
Post Base	36.33	0.23	36.40	23.43	1.57	24.00	131.80	/	86.50	8.04	/	9.04	131.50	/	83.83	73.17	8.33	73.50	230.30	54.84	255.00	41.12	41.83	20.75
Pre PPE	36.33	0.23	36.40	24.28	0.51	24.50	131.80	/	86.50	8.04	/	9.04	131.50	/	83.83	73.17	8.33	73.50	227.70	60.54	254.50	42.43	44.75	21.15
Post PPE	37.02	0.15	37.05	26.90	0.53	26.95	126.20	/	89.00	16.89	/	11.08	126.00	/	85.33	86.83	7.17	88.00	179.80	35.13	179.00	52.66	34.80	39.55
Pre Cool	36.30	0.49	36.50	24.50	0.46	24.50	130.80	/	82.50	15.34	/	6.13	123.00	/	83.00	78.67	14.31	88.00	228.70	59.52	255.50	41.83	43.81	20.50
Post Cool	36.40	0.32	36.55	25.83	0.68	26.00	127.80	/	82.00	10.46	/	11.10	127.00	/	81.50	75.17	7.65	77.50	231.50	59.83	259.00	40.40	43.79	18.60

The mean, standard deviation (SD) and median before and after the activity are shown

*avg* average, *bpm* beats per minute, *CPI* concentration performance indicator, *dia* diastolic, *SD* standard deviation, *sys* systolic, *TNE* total number of errors

## References

[1] Enander AE (1989). Effects of thermal stress on human performance. Scand J Work Environ Health.

[2] Verbeek JH, Rajamaki B, Ijaz S, Sauni R, Toomey E, Blackwood B, Tikka C, Ruotsalainen JH, Kilinc Balci FS (2020). Personal protective equipment for preventing highly infectious diseases due to exposure to contaminated body fluids in healthcare staff. Cochrane Database Syst Rev.

[3] Kucharski AJ, Russell TW, Diamond C (2020). Early dynamics of transmission and control of COVID-19: a mathematical modelling study. Lancet Infect Dis.

[4] Giunta RE, Frank K, Moellhoff N (2020). Die COVID-19 Pandemie und ihre Folgen für die Plastische Chirurgie und Handchirurgie. Handchir Mikrochir Plast Chir.

[5] Khan S, Siddique R, Ali A (2020). The spread of novel coronavirus has created an alarming situation worldwide. J Infect Public Health.

[6] Malhotra N, Gupta N, Ish S, Ish P (2020). COVID-19 in intensive care. Some necessary steps for health care workers. Monaldi Arch Chest Dis.

[7] Zhou F, Yu T, Du R (2020). Clinical course and risk factors for mortality of adult inpatients with COVID-19 in Wuhan, China: a retrospective cohort study. Lancet.

[8] López-Sánchez JI, Hancock PA (2018). Thermal effects on cognition: a new quantitative synthesis. Int. J. Hyperthermia.

[9] Saini R, Srivastava K, Agrawal S, Das RC (2017). Cognitive deficits due to thermal stress: an exploratory study on soldiers in deserts. Med J Armed Forces India.

[10] Amasuomo T, Amasuomo J (2016). Perceived thermal discomfort and stress behaviours affecting students’ learning in lecture theatres in the humid tropics. Buildings.

[11] Havenith G, den Hartog E, Martini S (2011). Heat stress in chemical protective clothing: porosity and vapour resistance. Ergonomics.

[12] Lee JKW, Kenefick RW, Cheuvront SN (2015). Novel cooling strategies for military training and operations. J Strength Cond Res.

[13] Langø T, Nesbakken R, Færevik H (2009). Cooling vest for improving surgeons’ thermal comfort: a multidisciplinary design project. Minim Invasive Ther Allied Technol.

[14] QRSKIN GmbH.. https://www.qrskin.com/home.html. Accessed 20 May 2020.

[15] Brickenkamp R, Schmidt-Atzert L, Liepmann D (2010). Test D2-Revision: Aufmerksamkeits- Und Konzentrationstest [D2 Test: Attention and Concentration Test].

[16] FLIR Systems.. https://www.flir.com/globalassets/imported-assets/document/flir-one-pro-series-datasheet.pdf. Accessed 20 May 2020.

[17] Rieger A, Stoll R, Kreuzfeld S, Behrens K, Weippert M (2014). Heart rate and heart rate variability as indirect markers of surgeons’ intraoperative stress. Int Arch Occup Environ Health.

[18] Schmit C, Hausswirth C, Le Meur Y, Duffield R (2017). Cognitive functioning and heat strain: performance responses and protective strategies. Sports Med.

[19] Berg RJ, Inaba K, Sullivan M (2015). The impact of heat stress on operative performance and cognitive function during simulated laparoscopic operative tasks. Surgery.

